# Case of colon perforation due to segmental absence of intestinal musculature accompanied by cancer treated with colonic resection and anastomosis

**DOI:** 10.1186/s40792-024-02050-1

**Published:** 2024-10-28

**Authors:** Eiki Sato, Yuki Seo, Yuta Matsukawa, Chang Shun-Kai, Masanori Kimura, Tomoko Takesue, Norihiro Kishida, Ikumi Hamano, Go Hoshino, Hideyuki Tokura, Takayuki Takahashi, Kazuhiko Shimizu

**Affiliations:** 1 Department of Surgery, Japanese Red Cross Ashikaga Hospital, Tochigi, Japan; 2 Department of Diagnostic Pathology, Japanese Red Cross Ashikaga Hospital, Tochigi, Japan

**Keywords:** Segmental absence of intestinal musculature, Carcinoma, Functional end-to-end anastomosis, Case report

## Abstract

**Background:**

Segmental absence of intestinal musculature (SAIM) is a partial defect of intestinal muscularis propria without diverticulum. Many reports indicate that the increase in intestinal pressure caused by enemas or endoscopic examinations leads to bowel perforation, but there are few reports involving malignant tumors. Moreover, few reports have had good outcomes after performing one-stage intestinal anastomosis.

**Case presentation:**

A 60-year-old male came to the office with right-side abdominal pain, and was diagnosed with acute generalized peritonitis caused by ascending colon perforation. Emergency laparotomy was performed, and oval and smooth perforation at the ascending colon was observed, which caused ascites with feces. In addition, there was a tumor on the distal side. The terminal ileum was not dilated, so the cause of the perforation was more likely the SAIM-related thin intestinal wall rather than increased internal intestinal pressure due to obstruction of the tumor. Therefore, a right hemicolectomy with functional end-to-end anastomosis (FEEA) between the ascending colon and ileum was performed, rather than creating a stoma. On pathological examination, the resected bowel segments had a partial defect of intestinal muscularis propria around the perforation, leading to the diagnosis of SAIM. The patient had a favorable postoperative course without anastomotic issues and was discharged safely.

**Conclusions:**

This case implies that initial intestinal anastomosis can be performed without creating a stoma when SAIM is suspected from the shape of the perforation and proximal intestine. This case report suggests surgeons should keep SAIM in mind during operations for colon perforations.

## Background

Segmental absence of intestinal musculature (SAIM) is characterized by partial or complete defects of intestinal muscularis propria without diverticulum, which results in intestinal perforation. First reported as a cause of intestinal obstruction and perforations in newborns [1–5], SAIM has also been reported in adults recently [6–17]. There are many reports that an increase in intraluminal pressure due to enemas or endoscopic examinations can cause bowel perforation, but to our knowledge, there have been few reports involving malignant tumors as so far. In addition, there are few reports of good outcomes after performing initial intestinal anastomosis, such as functional end-to-end anastomosis (FEEA), during surgery. This time, we report a case where, judging from intraoperative findings, we determined that the perforation of the colon was caused by SAIM on the proximal side of ascending colon cancer, and we were able to perform a one-stage intestinal anastomosis using FEEA.

## Case presentation

**Fig. 1:**
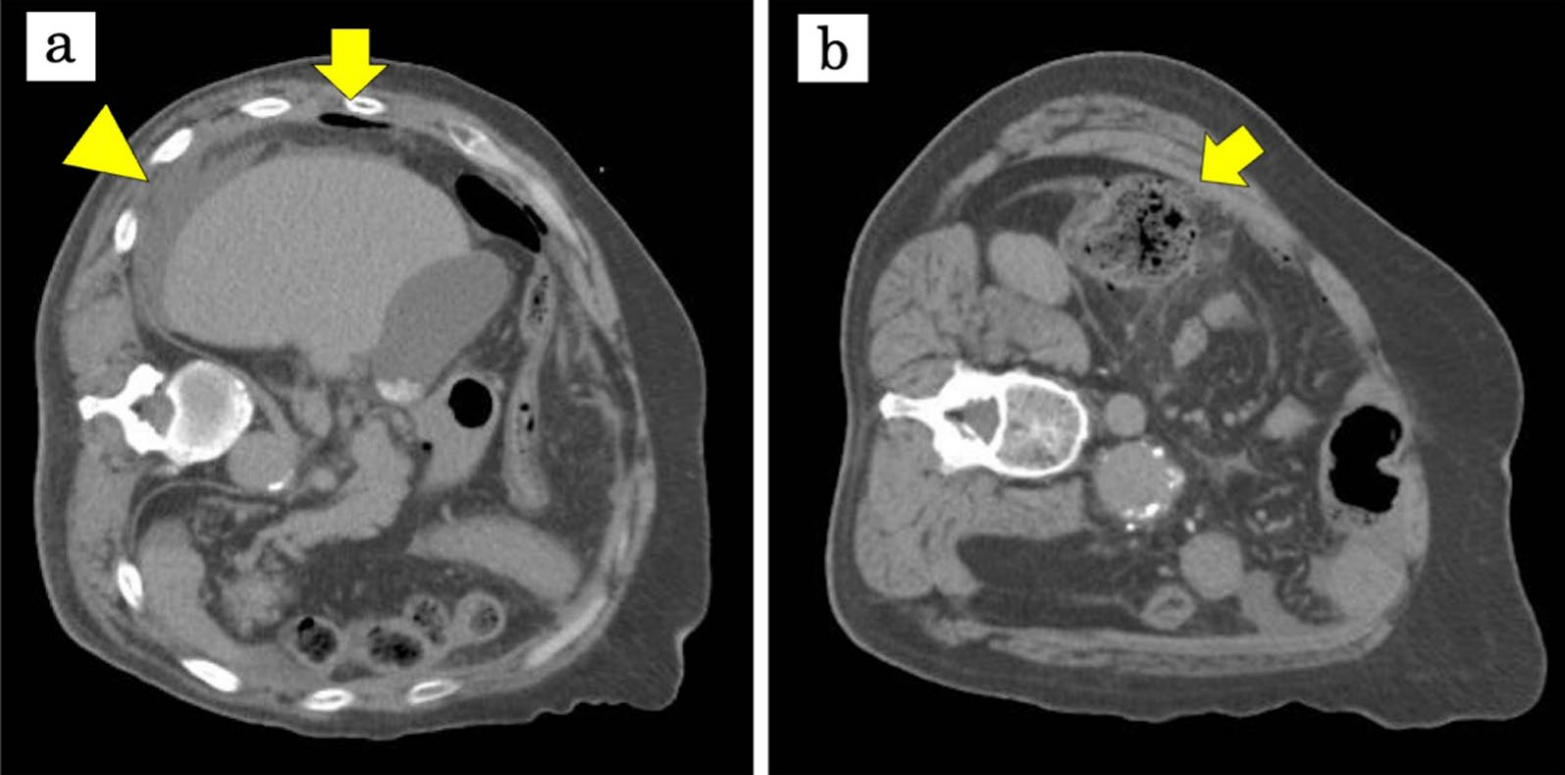
Abdominal computed tomography. During CT examination, the patient could not lie in the supine position due to severe abdominal pain and was scanned on the left decubitus position. CT revealed excessive free air (arrow) and ascites including stool below the liver (arrowhead) (**a**). The perforation (arrow) was on the proximal side of the mass (**b**). There are no apparent diverticula in CT images

A 60-year-old male presented with right flank pain, and was diagnosed with acute generalized peritonitis caused by ascending colon perforation. He suffered abdominal aortic aneurysm rupture and had undergone Y-grafting and open abdominal management in the past. Physical examination showed severe tenderness to palpitation with rebound and guarding. Computed tomography (CT) revealed a mass in the ascending colon with excessive free air and ascites including stool below the liver (Fig. 1).

**Fig. 2:**
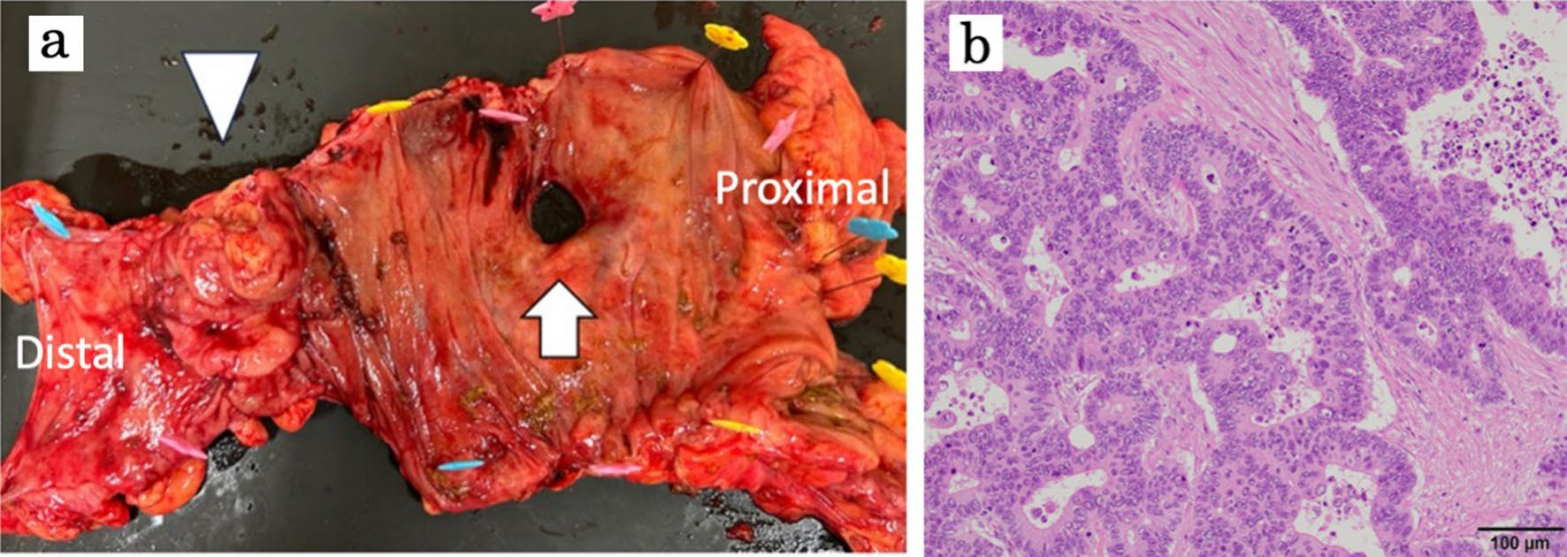
Gross and microscopic findings of the resected tumor specimen. The resected specimen shows the entire circumstance of a type II tumor in the ascending colon (arrowhead), featuring an oval-shaped perforation with a smooth border on the proximal side of the tumor (arrow) (**a**). The ascending colon tumor was identified as a moderately differentiated adenocarcinoma with metastasis to one lymph node(1/2)–pT3 pN1a cM0 stage IIIB according to the TNM staging of AJCC Cancer Staging 8th Edition (HE stain; original magnification × 200) (**b**)

Emergency laparotomy was performed through an abdominal incision along the previous surgical scar. Reaching the abdominal cavity, there were dark red turbid ascites with a foul odor. After sucking out the ascitic fluid as much as possible, an oval and smooth perforation at the ascending colon was observed. In addition, there was an extraserosal tumor on the distal side (Fig. 2). The terminal ileum was not dilated, so it was assumed that the cause of the perforation was not merely the increased internal intestinal pressure due to tumor obstruction. Considering the form of the perforation, it was highly likely that the internal intestinal pressure was exerted on the thin intestinal wall by SAIM, which led to the perforation.

**Fig. 3:**
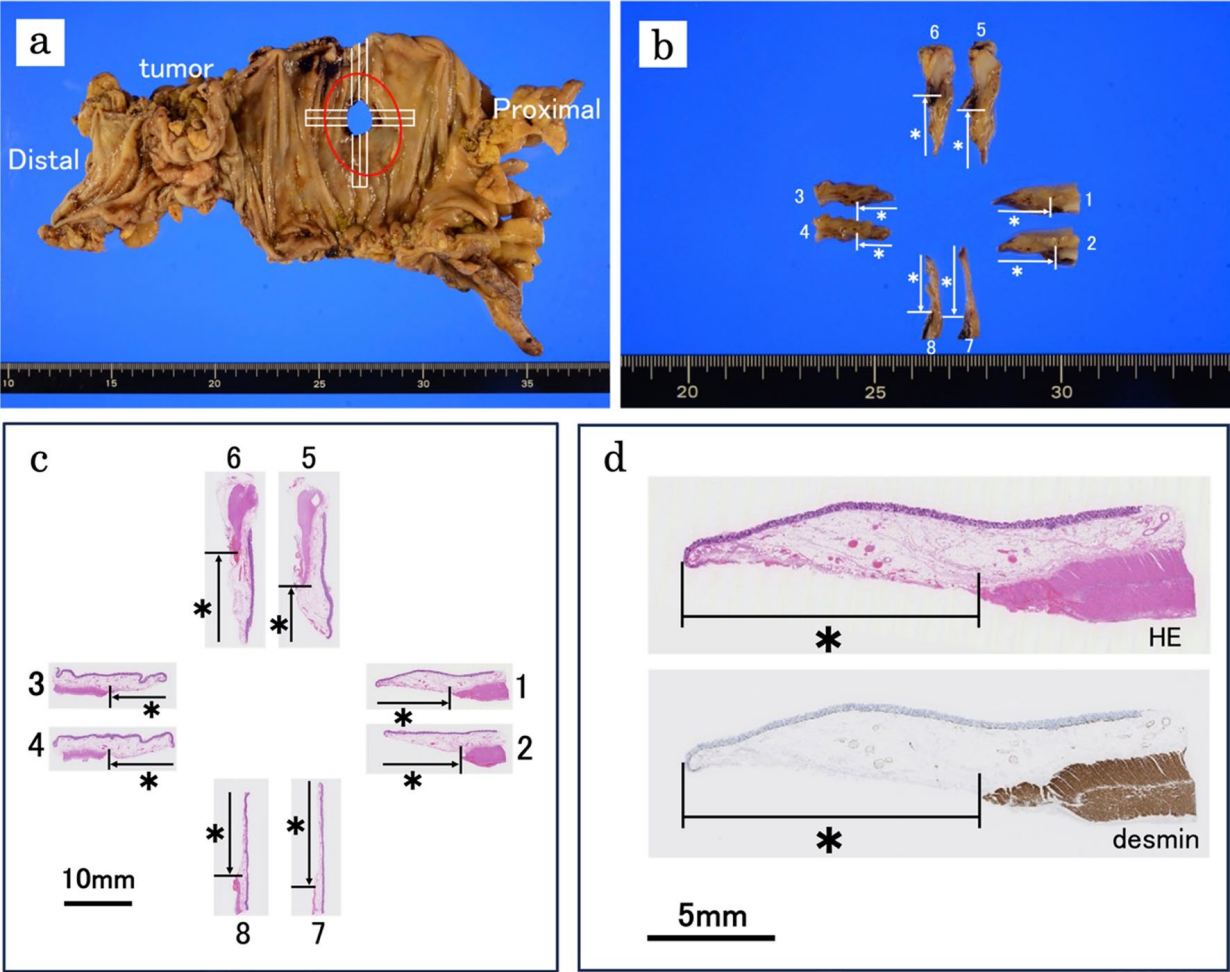
Gross and microscopic findings of SAIM. The perforation site was cut crosswise on the specimen fixed in formalin (**a**, **b**). Images** c**,** d** show the gross appearance and microscopic findings of the cut surface. Each sample (number 1–8) shows a partial defect of muscularis propria (HE stain; original magnification × 12.5) (**c**). When using desmin stain, which stains only smooth muscle, the muscularis propria was stained brown, and the defect of the intestinal muscularis propria was emphasized (desmin stain; original magnification × 12.5) (**d**, lower part)

Therefore, we chose to perform a right hemicolectomy with FEEA between the ascending colon and ileum was performed, avoiding creating a stoma. The lymph node dissection was limited to D2 dissection to shorten the operation time. On pathological examination, the mass on the ascending colon was a moderately differentiated adenocarcinoma with one lymph node metastasis (Fig. 2). In addition, resected bowel segments had a partial defect of intestinal muscularis propria around the perforation, leading to the diagnosis of SAIM (Fig. 3). The patient only experienced complications up to Clavien–Dindo classification Grade II, with no anastomotic issues, and was discharged in good condition on postoperative day 31.

## Discussion

SAIM, a partial defect of the intrinsic muscular layer of the intestine, was first reported by Emanuel et al. in 1967 as a cause of neonatal intestinal obstruction [1]. There are two possible causes of SAIM in neonates: one is abnormal intestinal development during the fetal period, and the other is intestinal ischemia after birth [2, 3]. In recent years, SAIM has also been reported in adults, where it was identified following bowel perforation, in addition to the previously reported neonatal cases. The cause of SAIM in adults is still unclear. The absence of muscle that appears in adults is believed to originate during the development of the fetal intestinal tract, persist into adulthood, and only become apparent when intestinal perforation occurs. However, there is a report that 38% of adults with SAIM had a history of intestinal ischemia, such as hypertension, vascular disorders, or chronic constipation, suggesting that intestinal ischemia may be a trigger [6].

SAIM is most often detected after performing surgery for intestinal perforation and diagnosed by the resected specimen pathologically. We searched for adult cases of SAIM in PubMed using the keywords “Segmental absence of intestinal musculature” from 1967 to 2023, as summarized in Table 1. While the causes of intestinal perforation related to SAIM are often unknown, most reports are due to factors associated with increased intestinal pressure such as endoscopic examinations, constipation, and enemas. Tsuyuki et al. retrospectively examined 109 cases of intestinal perforation and found that SAIM was the second most frequent cause of intestinal perforation (26 cases, 24%) [7]. This study suggests that many cases of intestinal perforation previously diagnosed as idiopathic may have been caused by SAIM, and now it is hoped that SAIM will become widely recognized among surgeons and pathologists. In addition, there are very few reports of cases involving malignant tumors, as seen in Table 1 below.

**Table 1:** Adult cases of SAIM reported so far

Case #	Authors/year	Age (year)	Sex	Cause of perforation	Location	Treatment
1	Darcha et al. [8], 1997	64	Female	Iatrogenic colonic perforation during laparoscopic polypectomy	Sigmoid colon	Unknown
2	Tawfiq et al. [9], 1998	34	Male	Unknown/idiopathic	Jejunum	Conservative therapy for 2 days, and exploratory laparotomy Resection of small bowel and end-to-end anastomosis
3	Aldalati et al. [10], 2009	Middle age	Male	No perforation	Jejunum	Resection
4	Procházka et al. [11], 2010	28	Female	Unknown/idiopathic	Ascending colon	Section and right-sided hemicolectomy
5–11	Tamai er al. [12], 2013	44–89 Mean 63.3 Median 61	Female 4 Male 3	Unknown/idiopathic	Jejunum 2 Ileum 1 Ascending colon 1 Sigmoid colon 3	Unknown
12	Nandedkar et al. [13], 2015	48	Male	Unknown/idiopathic	Small bowel	Resection anastomosis
13	Rewhorn et al. [14], 2015	68	Female	Unknown/idiopathic	Sigmoid colon	Unknown
14	Nawar and Sawyer et al. [6], 2016	64	Female	Constipation	Descending colon	Left hemicolectomy Colon anastomosis
15	Tseng et al. [15], 2016	60	Female	Unknown/idiopathic	Sigmoid colon	Hartmann procedure
16	Shibata et al. [16], 2023	58	Male	Unknown/idiopathic	Sigmoid colon	Hartmann procedure
17	Funaki et al. [17], 2023	30's	Male	Vascular Ehlers–Danlos syndrome	Sigmoid colon	Hartmann procedure
18	Our case	60	Male	Colon cancer	Ascending colon	Right hemicolectomy with FEEA

In previous reports, the pathological findings of SAIM were accompanied by a "punched out" form of perforation, with no significant infiltration or degeneration of inflammatory cells associated with the defect in the surrounding mucosa [12]. In this case, in the same way, there was a perforation with well-defined margins and no surrounding inflammatory cell infiltration. Although hemorrhage was observed on the serosal surface at the margin of the perforated area, there was little evidence of ischemic degeneration or necrosis in the muscularis propria. Additionally, no cancer invasion or diverticula were observed around the perforated area.

In our case, intraoperative findings revealed a tumor suspected to be advanced cancer in the ascending colon, with an oval and smooth perforation on the proximal side of the tumor. In cases of a proximal perforation from a cancer, the first mechanism usually considered is an increase in intraluminal pressure on the proximal side due to tumor-induced intestinal obstruction, leading to wall rupture. In such cases, the proximal side of the intestine is often still dilated and edematous, and the surgical approach strategy often involves avoiding anastomosis by creating stoma or double-barrel ileostomy on the proximal side after anastomosis. In our case, the dilation on the proximal of the perforation was mild, and no dilation of the terminal ileum was observed, suggesting that the perforation was more likely caused by a slight increase in intraluminal pressure on the thin intestinal wall due to SAIM rather than a typical wall rupture due to increased intraluminal pressure caused by obstruction from the ascending colon cancer. Therefore, we avoided a stoma and performed intestinal resection including the abscess and perforation site, and a one-stage intestinal anastomosis using FEEA. There are only a few cases in which a one-stage bowel anastomosis has been performed in patients with generalized peritonitis. In many such cases, inflammation has spread to the intestines, resulting in edema and degeneration of the intestinal wall. Consequently, a one-stage intestinal anastomosis is often not performed; instead, a stoma is created to rest the intestines and reduce pressure within them. However, in SAIM cases like this one, when a perforation is observed without intestinal edema, degeneration, or necrosis due to ischemia, a one-stage intestinal anastomosis may be considered as an option.

In such a case, it is also necessary to consider the patient's co-morbidities, such as diabetes mellitus and malnutrition. Intraperitoneal lavage and drainage are effective for generalized peritonitis, and these procedures have been performed in this case as well.

## Conclusions

We have reported a case in which we judged from surgical findings that FEEA could be performed for generalized peritonitis caused by a perforated ascending colon cancer accompanied by SAIM, and successfully saved the patient. This case suggests that by keeping the possibility of SAIM in mind based on the shape of the perforation and the degree of bowel dilation observed during surgery, it may be possible to proactively perform one-stage intestinal anastomosis even in cases of colon perforation.

## Data Availability

All data supporting our findings are contained within the manuscript.

## Abbreviations

SAIM: Segmental absence of intestinal musculature
FEEA: Functional end-to-end anastomosis
CT: Computed tomography
